# Cryptic diversity and diversification processes in three cis-Andean *Rhamdia* species (Siluriformes: Heptapteridae) revealed by DNA barcoding

**DOI:** 10.1590/1678-4685-GMB-2020-0470

**Published:** 2021-07-12

**Authors:** Josiane Ribolli, Evoy Zaniboni, Bianca Maria Soares Scaranto, Oscar Akio Shibatta, Carolina Barros Machado

**Affiliations:** 1 Universidade Federal de Santa Catarina, Departamento de Aquicultura, Lagoa do Peri, Laboratório de Biologia e Cultivo de Peixes de Água Doce, Florianópolis, SC, Brazil.; 2 Universidade Estadual de Londrina, Departamento de Biologia Animal e Vegetal, Centro de Ciências Biológicas, Londrina, PR, Brazil.; 3 Universidade Federal de São Carlos, Departamento de Genética e Evolução, São Carlos, SP, Brazil.

**Keywords:** Aquaculture, freshwater fishes, marine transgression/regression, phylogeography, underestimated diversity

## Abstract

The wide distribution of the Neotropical freshwater catfish *Rhamdia* offers an excellent opportunity to investigate the historical processes responsible for modeling South America’s hydrogeological structure. We used sequences from cis-Andean and Mesoamerican *Rhamdia* species to reconstruct and estimate divergence times among cis-Andean lineages, correlating the results with known geological events. Species delimitation methods based on distance (DNA barcoding and BIN) and coalescence (GMYC) approaches identified nine well-supported lineages from the cis-Andean region from sequences available in the BOLD dataset. The cis-Andean *Rhamdia* lineages diversification process began in Eocene and represented the split between cis-Andean and Mesoamerican clades. The cis-Andean clade contains two principal groups: Northwest clade (MOTUs from Amazon, Essequibo, Paraguay, and Itapecuru basins) and Southeast clade (Eastern Brazilian shield basins (Paraná, Uruguay, Iguaçu, and São Francisco) plus eastern coastal basins). The diversification of the cis-Andean *Rhamdia* lineages results from vicariance and geodispersion events, which played a key role in the current intricate distribution pattern of the *Rhamdia* lineages. The wide geographical distribution and large size of the specimens make it attractive to cultivate in different countries of the Neotropical region. The lineages delimitation minimizes identification mistakes, unintentional crossings by aquaculture, and reduces natural stocks contamination.

## Introduction

Neotropical freshwater fishes constitute a host of global biodiversity (Albert et al., 2020), and more recent surveys have described at least 6,080 species (Dagosta and de Pinna, 2019). However, there is a consensus that this number is underestimated, with innumerable new species being described each year (Reis et al., 2016; Dagosta and de Pinna, 2019). The vast Neotropical environments allow ecological species adaptations (Cooke et al., 2012), which when they occur widely and in different habitats (e.g., large and rushing rivers, caves, lakes, ponds, and streams) make it difficult to carry out a broad sampling, thus contributing to hiding species that were historically identified and delimited only by traditional taxonomy (Melo et al., 2016). Beyond this enormous habitat variety, phenotypic plasticity and cryptic species’ occurrence are the main reasons for these underestimating (Bickford et al., 2007). 

The correct species identification is essential for different areas, such as aquaculture, biodiversity conservation, biogeography, ecology, comparative biology, and systematics (e.g. Agapow et al., 2004; Bortolus, 2008; Maclaurin and Sterelny, 2008; Frankham et al., 2012; Scaranto et al., 2018). DNA barcoding is a molecular tool widely used to delimitate and identify cryptic species in the most varied taxonomic groups (Hebert et al., 2003; Hebert *et al.*, 2004). An alternative approach to practical help correctly identifies the species when morphological traits are insufficient. This method, associated with a quantitative species delimitation approach (GMYC - Pons et al., 2006; Fujisawa and Barraclough, 2013), have been employed with success in the vast number of Neotropical fish species (Pereira et al., 2013; Machado et al., 2017; Ramirez et al., 2017). This approach allows the identification of Operational Molecular Taxonomy Units (hereafter named MOTUs) and species resolution with precision (Machado *et al.*, 2017; Melo et al., 2018). 

*Rhamdia* is an emblematic genus with an intriguing taxonomic scenario and species delimitation issues (Albert et al., 2020; Ríos et al., 2020). In Brazil’s southern region, there is a great interest in the commercial cultivation of *Rhamdia quelen* (Battisti et al., 2020). Its broad geographical distribution and low temperatures tolerance make it attractive to cultivate in different parts of the Neotropical region, especially in Southern Brazil, Uruguay and Argentina (Salhia *et al*., 2008; Gomes et al., 2000; Ittzéz *et al*., 2020). Thus, it is necessary to identify the species to avoid unintentional crossings (Scaranto et al., 2018). More recently, some synonyms of *R. quelen* were recovered with integrative methods (Garavello and Shibatta, 2016; Ribolli et al., 2017), new species were described (DoNascimiento et al., 2004; Bichuette and Trajano, 2005; Angrizani and Malabarba, 2018), flagging *Rhamdia quelen* as a species complex. Koerber and Reis (2020) suggested a new type-locality in an affluent of the Guanabara Bay, state of Rio de Janeiro, to replace a missing type specimen of *Rhamdia quelen*. However, a taxonomic revision of *Rhamdia* is straightaway necessary to explain species delimitation problems, which hinder their correct identification (Koerber and Reis, 2020). Because *Rhamdia* species occur in almost every freshwater environment of the Neotropical region, an accurate dataset (e.g., BOLD system database) helps understand its distribution. Therefore, precise information on how *Rhamdia* lineages is distributed in the main geographic basins of the cis-Andean region offers an excellent opportunity to investigate the historical processes responsible for modeling the hydrogeological structure of South America, and will contribute to studies on the identification of lineages and species that still make up the *Rhamdia quelen* species complex.

In this study, we used species delimitation methods based on distance (DNA barcoding and BIN), and coalescence (GMYC) approaches to identify genetic units of three *Rhamdia* species from the cis-Andean region. The aim is to point out the independent evolutionary lineages flagging potentially undescribed taxa. Although we are aware of the limitations to create biogeographic hypotheses based on single-locus, we also investigated which historical events were responsible for diversification processes of *Rhamdia* lineages. Based on the evolution of modern drainage systems and biogeographic history of Neotropical freshwater fish species from the major basins of South America (e.g., Montoya-Burgos, 2003; Hubert and Renno, 2006; Ramirez et al., 2017), we expect that vicariance and geodispersion events were driven diversification processes. 

## Material and Methods

### Sampling

Fin clips of 36 specimens morphologically identified as *Rhamdia quelen* complex*, Rhamdia branneri*, and *Rhamdia voulezi* were sampled from 2014 to 2018. Specimens were collected from the Uruguay, Benedito Novo and Itapocu rivers, and Peri Lagoon, because this species are important as aquaculture resource in this region. Tissue samples were preserved in 95% ethanol and stored at -20 ^o^C. Individuals sampled were photographed, stored in the collection of the Laboratório de Biologia e Cultivo de Peixes de Água Doce (LAPAD/UFSC), Santa Catarina State, Brazil. Collecting license was provided by Instituto Brasileiro do Meio Ambiente e dos Recursos Naturais Renováveis - IBAMA (16/2011 and 659/2015). Vouchers were deposited in the ichthyological collection of the Museu de Zoologia of the Universidade Estadual de Londrina (MZUEL).

**Figure 1 -:**
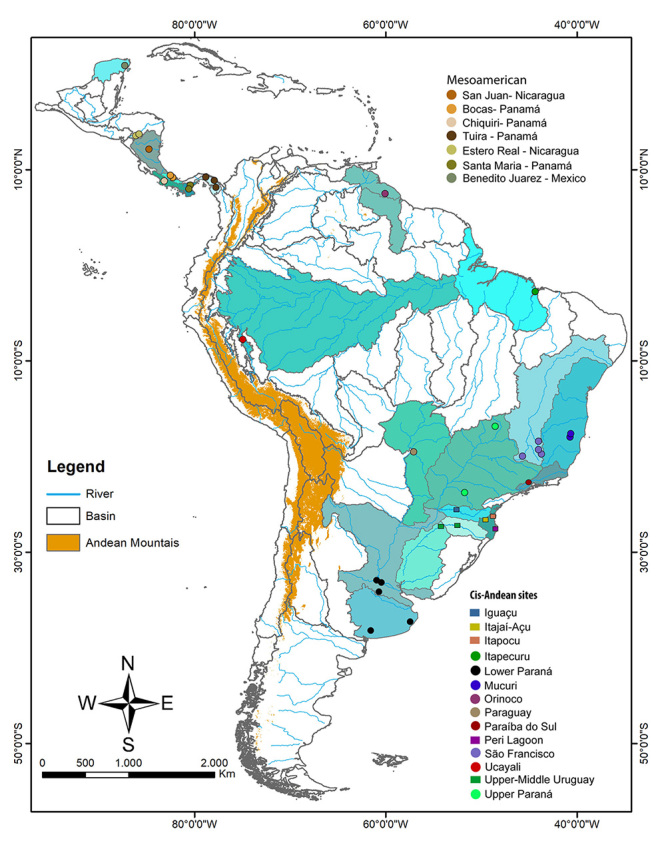
Sampling sites of *Rhamdia* species in South America. Circles represent samples from BOLD System (http://www.boldsystems.org/), while squares are sequences obtained in this study. Colors indicated the hydrographic basin where collected.

We also included 63 barcodes of *Rhamdia* genus from the cis-Andean region, corresponding to Itapecuru, Paraná, Mucuri, Essequibo, Paraguay, Paraíba do Sul, São Francisco, and Ucayali basins; four sequences of *R. laticauda* from Nicaragua and Panama; eight sequences of *R. quelen* from Panama and Nicaragua (code BSFFA) and three sequences of *R. guatemalensis* from Mexico (code HBGM), both from the Mesoamerican region; and one sequence of *Pimelodella* sp. as outgroup were downloaded from Barcode of Life Database (BOLD; www.boldsystems.org). We used exclusively (except the Paraguay River basin samples from Genbank) sequences from the BOLD system because this database is accurate and allows verification of the sequences’ quality (trace files). Because *Rhamdia* is widely distributed, we took care to evaluate all available sequences, reducing the mistakes that may be associated with public and non-generated sequences; as such, we do not use most Genbank and some BOLD sequences. Details like voucher number, locality information, and accession numbers for databases are shown in Supplementary Table S1. Our final dataset for species delimitation and phylogeographic analyses was composed of 115 specimens distributed for all South America (Figure 1 and Table S1).

### DNA amplification and sequencing

*Rhamdia* specimens’ DNA was extracted from caudal fin fragments using a salt method (Aljanabi and Martinez, 1997). COI fragments were amplified by polymerase chain reaction (PCR) using primers FishF1 and FishR1 (Ward et al., 2005), according to Bellafronte et al. (2013). After confirming amplification in a 1% agarose gel, the PCR products were purified with 20% PEG (Lis, 1980). Sequencing reactions were performed using BigDye TM Terminator v 3.1 (Cycle Sequencing Ready Reaction Kit, Applied Biosystems), and fragments were sequenced in ABI 3500XL (Applied Biosystems). All sequences were deposited in BOLD (accession numbers are given in Table S1 - PBRH code).

### Data analysis

The sequences were aligned, using the Clustal W algorithm, and edited in Geneious R7 6.1.6 (http://www.geneious.com, Kearse et al., 2012). As mentioned, we also combined sequences available in the BOLD system (http://www.boldsystems.org): cis-Andean *Rhamdia* specimens (codes FARG, LARI, FPSR, MUCU, BSB, FUPR, ITAPE, PBRH, BSFFA, ANGBF, and HBGM), Mesoamerican *Rhamdia* specimens (codes HBGM and TZGAA), *Pimelodella* sp. (code HM) as the outgroup, and six *Rhamdia* sequences available in Genbank (code KU).

Because of the uncertain taxonomic validity of *Rhamdia* species, mainly *R. quelen* complex, we used a phylogenetic General Mixed Yule Coalescent (GMYC) approach based on single-locus data to estimate the MOTUs in the present dataset. An ultrametric gene tree required by the analysis was generated in BEAST v.2.5.2 (Bouckaert et al., 2014), with HKY+G substitution model calculated in the jModelTest 2.1.4 (Darriba et al., 2012) using the Bayesian Information Criterion, relaxed molecular clock with a lognormal distribution, and Birth and Death model as tree prior. The other priors used in this analysis were kept in default. Three independent runs were carried out with 50 million generations each. All runs were combined using LogCombiner v.2.5.2, and 25% of the generations were discarded as burn-in. The posterior sample of trees was summarized in the TreeAnnotator to produce a maximum clade credibility tree. The effective sample size for all parameters (ESS) was verified in Tracer v1.5 (Rambaut and Drummond, 2007). GMYC analysis was carried out in SPLITs (SPecies Limits by Threshold Statistics; Monaghan et al., 2009) package with RStudio (http://r-forge.r-project.org/ projects/ splits), using the unique threshold method to detect the transition point between intra- and interspecific relationships. 

We also employed a distance approach to determine the MOTUs present in our dataset (DNA barcoding methodology - Hebert et al., 2003). According to this method, sequences from the same species show low genetic distance than the sequences from different species given a threshold. Because we are focused on determining the lineages from the cis-Andean region, we estimated an optimum threshold (OT) for this specific dataset. To establish the OT, we used the localMimima function from the SPIDER package (SPecies IDentity and Evolution in R, Brown et al., 2012) in the R platform. This function is based on the concept of the barcoding gap (Hebert *et al.,* 2003) and used a Kimura 2-Parameter (K2P) distance matrix to find the transition between intra- and interspecific distances. The method does not require a priori information about taxon identity. After the OT was determined, we used jMOTU (Jones *et al*., 2011) to obtain the MOTUs. We also employed the Barcode Index Number system (BIN - Ratnasingham and Hebert, 2013), an online framework based on the distance that cluster barcode sequences algorithmically. 

It is possible to find incongruous results among methods because they employ distinct algorithms (Ramirez *et al.*, 2020). Thus, the final adopted species delimitation model was defined from consensus across methods and the low number of singletons, MOTUs composed of only one sequence. Genetic distances within and between MOTUs were estimated using Mega 7.0 (Tamura et al., 2013) based on the K2P evolution model, with 1,000 replicates bootstraps. To elucidate relationships between haplotypes, we also reconstructed a haplotype network using PopART v. 1.7 (Leigh and Bryant, 2015) through the median-joining distance method (Bandelt et al., 1999). 

To comprehend which historical processes were responsible for MOTUs diversification in the cis-Andean *Rhamdia* genus, we estimated divergence time and associated changes in hydrogeological landscapes. The divergence time analysis was also estimated in BEAST v.2.5.2 using a fossil record. Because there is no fossil record for the *Rhamdia* or its family (Heptapiteridae), calibration points were chosen based on taxa from Pimelodidae: *Phractocephalus hemioliopterus* (Lundberg and Aguilera, 2003) and *Steindachneridion iheringi* (Hardman and Lundberg, 2006). Therefore, we added to our dataset sequences of *P. hemioliopterus*, *S. scriptum,* and *S. parahybae* from the BOLD system (Table S1). We placed the fossils as a most recent common ancestor - MRCA - of each genus (crown-group). We set the calibration points with lognormal priors. Specifically, for *P. hemioliopterus,* we implemented an offset to 9 Mya, with a mean of 0.2 and standard deviation of 1.25 (95%: 9.11 - 18.5 Mya), while for *Steindachneridion* clade, the offset was 22 Mya, with mean 0.2 and standard deviation of 1.2 (95%: 22.2 - 30.8 Mya). The Bayesian topology was performed following the same parameters described for the GMYC analysis. The mean and 95% highest posterior density (HPD) estimates of divergence times and inferred clades’ posterior probabilities were visualized using the software FigTree v.1.3.1 (http://tree.bio.ed.ac.uk/software/figtree/).

## Results

This study’s barcode sequences showed more than 550 base pairs (only MNCE was removed because it showed 465 pb). Stop codons, insertions, or deletions were not observed. After alignment and edition, the final matrix had 652 characters, of which 500 positions were conserved, 152 were variable, and 122 were parsimony-informative sites. The sequences of the Jequitinhonha River basin (Code JEQUI) were not added to the dataset, because in Pugedo et al. (2016), the sequences are interspersed among other genera, such as *Pimelodella*, *Steindachneridion,* and *Colossoma*. Considering this situation and the complexity of the *Rhamdia* genus, we chose not to use these data. However, we present an additional tree (Figure S1), generated with the code sequences JEQUI and RDOCE.

**Figure 2 -:**
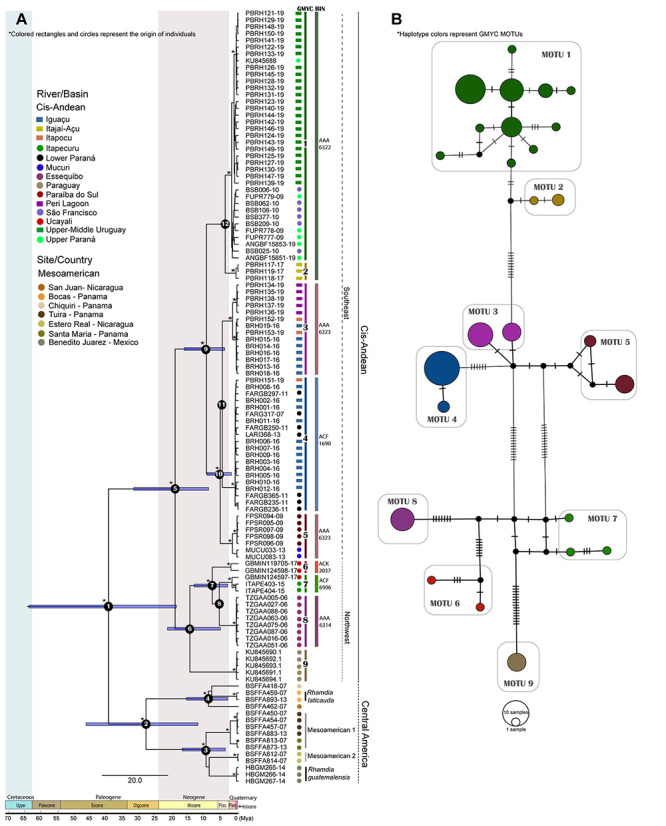
Time-calibrated topology of Mesoamerica and cis-Andean *Rhamdia* with species delimitation analyses (A) and median-joining haplotype network for cis-Andean *Rhamdia* (B) based on COI sequences. Node numbers represent the diversification events, and asterisks represent posterior probabilities above 0.9. Circles represent samples from BOLD System (http://www.boldsystems.org/), while squares are sequences obtained in this study. In the haplotype network, the circle size is proportional to the number of sequences. There is not sharing haplotypes among MOTUS. Black circles indicate haplotypes not sampled.

The likelihood of the GMYC model in the species delimitation approach was significantly superior (L=319.5186) than the likelihood of the null model (L_0_=300.0984, p<0.000), rejecting the null hypothesis that all individuals belong to a single MOTU. The GMYC model determined 16 MOTUs (confidence interval, 15-48). As expected, the *R. quelen* complex from cis-Andean basins was polyphyletic. The species is represented by more than one MOTU (7 MOTUs widely distributed in major cis-Andean basins of South America MOTUs 1, 2, 5, 6, 7, 8 and 9), flagging the presence of cryptic species or a severe taxonomic problem (Figure 2A). The endemic species from the Iguaçu River (*R. voulezi* and *R. branneri*) were composed of one MOTU each, respectively, MOTUs 3 and 4. Although the latter also have specimens identified as *R. quelen*, probably they represent misidentifications, once *R. branneri* was recognized by molecular and morphological analysis (Garavello and Shibatta, 2016; Ribolli et al., 2017). In the haplotype network analysis, all estimated *Rhamdia* MOTUs from the Cis-Andean region are well-separated in distinct haplogroups (Figure 2B). Among Mesoamerican species, only *R. guatemalensis* was represented by one MOTU. *Rhamdia quelen* (named *Rhamdia* Mesoamerican 1 and 2) and *R. laticauda* showed a hidden genetic diversity that also needs to be assessed. However, because the sampling was small, we will not focus on this issue in the present study.

The estimated OT of the cis-Andean dataset was 0.91%. Ten MOTUs were identified using the OT value as a barcode threshold in jMOTU software. The OT showed congruence with GMYC analysis for cis-Andean MOTUs, except for MOTU Ucayali (two specimens from Ucayali basin), which break-up into two MOTUs forming singletons. The BIN results revealed six molecular units: AAA6222 (correspondent to MOTUs 1 and 2), AAA6323 (MOTUs 3 and 5), ACF1690 (MOTU 4), ACK2037 (MOTU 6), ACF6906 (MOTU7), and AAA6314 (MOTU 8) (see Figure 2A). The sequences from GenBank (code KU, from Paraguay Basin) do not have a BIN. 

GMYC results was considered the best species delimitation model based on congruence among different methods and a low number of singletons. The mean intra-MOTUs genetic distances were lower than the optimum threshold (0.91%), while all mean inter-MOTUs genetic distances were higher than the threshold. These results were expected by DNA barcoding analysis and supporting the MOTUs estimated by GMYC analysis (Table 1).

**Table 1 -:** The mean intra-MOTU (in bold) and inter-MOTU genetic distances of *Rhamdia* lineages are based on the COI gene and the K2P model. Values are presented in percentage. UPUSF (Upper Paraná-Uruguay-São Francisco basins); IPLII (Iguaçu (*R. voulezi*)-Peri Lagoon-Itapocu); ILPI (Iguaçu *(R. branneri)*-Lower Paraná-Itapocu); MOTU Mesoamerican (MOTU Mesoamerican 1 and Mesoamerican 2).

		1	2	3	4	5	6	7	8	9	10	11	12	13
Cis-Andean MOTUs	1. MOTU 1 (UPUSF)	**0.33**												
	2. MOTU 2 (Itajaí-Açu)	1.24	**0.10**											
	3. MOTU 3 (IPLII)	2.99	2.25	**0.23**										
	4. MOTU 4 (ILPI)	3.90	3.13	1.48	**0.04**									
	5. MOTU 5 (Paraíba do Sul)	2.87	2.91	1.25	1.74	**0.27**								
	6. MOTU 6 (Ucayali)	5.46	5.26	4.53	5.30	4.72	**0.93**							
	7. MOTU 7 (Itapecuru)	4.85	4.87	4.07	4.67	3.98	2.59	**0.69**						
	8. MOTU 8 (Essequibo)	5.87	5.43	4.70	5.47	4.89	2.52	2.32	**0.00**					
	9. MOTU 9 (Paraguay)	5.77	5.27	3.51	3.96	3.95	4.16	3.31	4.16	**0.00**				
Mesoamerican MOTUs	10. MOTU Mesoamerican	10.10	10.19	9.30	10.24	9.12	9.42	9.63	9.47	8.46	**1.65**			
	11. *R. laticauda*	9.85	9.84	8.82	9.37	8.50	9.48	9.69	9.48	8.94	8.48	**2.32**		
	12. *R. guatemalensis*	9.25	9.28	8.40	8.98	7.76	8.96	9.42	10.11	7.64	3.15	8.20	**0.11**	
	13. *Pimelodella* sp.	12.38	11.98	10.87	11.31	11.21	11.88	11.76	11.14	10.78	13.47	11.59	12.88	-

The phylogenetic gene tree strongly supported almost all relationships among MOTUs (Figure 2A). Two main clades were observed in the *Rhamdia* genus: one composed of MOTUs from Mesoamerica basins, and the other comprised MOTUs from cis-Andean basins. The cis-Andean clade contains two principal groups: The Northwest clade that corresponds to the MOTUs from Amazon, Essequibo, Paraguay, and Itapecuru basins, and the Southeast clade composed of Eastern Brazilian shield basins (Paraná, Uruguay, Iguaçu and São Francisco) plus eastern coastal basins. 

The Southeast clade is also subdivided into two main groups well-supported: specimens from Upper Paraná/Upper and Middle Uruguay/São Francisco Basins (named here as MOTU 1) and from Itajaí-Açu basin (MOTU 2); and others composed by valid species from Iguaçu River (MOTU 3 and 4, *R. voulezi* and *R. branneri* respectively), and Paraíba do Sul (MOTU 5 - specimens from Paraíba do Sul and Mucuri rivers). The MOTU 3 also comprises individuals from Iguaçu and Peri Lagoon (Coastal Lagoon in Santa Catarina Island) basins, while MOTU 4 clade corresponds to individuals from Iguaçu, Itapocu, and Lower Paraná basins.

The Northwest clade comprises four MOTUs: individuals from Ucayali River, Upper Amazon basin (MOTU 6), specimens from Itapecuru river, Maranhense Gulf (MOTU 7), individuals from Barama River, Essequibo basin (MOTU 8), and specimens from Paraguay River basin (MOTU 9; Figure 2A).

The time divergence analysis suggests that the diversification processes in the *Rhamdia* genus began in the Eocene (Figure 2A, Figure S2). The *Rhamdia* genus’ most recent common ancestor (MRCA) was placed in 39.55 million years ago (95% HPD = 18.8 - 63.7 million years ago, Mya) and represents the split between cis- and trans-Andean clades. The MRCA cis-Andean MOTUs were dated at 19.2 Mya (95% HPD = 9.1 - 31.9 Mya) during Middle Miocene. All remaining diversification processes among cis-Andean MOTUs happened during the Miocene-Pleistocene epochs (Figure 2A).

## Discussion

### Molecular species delimitation of *Rhamdia* genus: underestimation diversity in the species complex

In our study, the species delimitation approach based on molecular data revealed a hidden diversity in the *Rhamdia quelen* complex*.* The results support the presence of at least six lineages from cis-Andean hydrographic basins depending on the used species delimitation approach (distance or phylogenetic and coalescent methods). Considering our parameters to choose the best species delimitation model and the phylogenetic species concept based on monophyly (Rosen, 1978), the BIN result (six MOTUs) was discarded. Although the OT also uses a clustering approach based on genetic distance, the high threshold employed by BIN (2.2% - Ratnasingham and Hebert, 2013) merges four allopatric MOTUs into two groups (AAA6322: MOTU 1 and 2; AAA6323: MOTU 3 and 5). The mean genetic distance above the OT between the clustered MOTUs (1 and 2; 3 and 5) flags that the BIN method underestimated the divergence of *Rhamdia* lineages. In future studies, its use as a species delimitation method for *Rhamdia* needs to be carefully evaluated.

The GMYC and distance analysis based on OT showed congruent results, except for specimens from Ucayali. According to the distance analysis (OT), each specimen represents one different MOTU. Talavera et al. (2013) argue that the presence of a high number of singletons could considerably reduce the result’s biological meaningfulness. Thus, we view this result as unlikely for now, but it is necessary to ascertain the presence of *Rhamdia* divergent lineages from the Amazonas region.

The GMYC has been used in numerous empirical studies (Machado et al., 2017; Ramirez et al., 2017, 2020; Melo et al., 2018), and simulation and empirical data suggest that the method is robust to different assumptions (Esselstyn et al., 2012; Reid and Carstens, 2012; Talavera et al., 2013). The method detected nine lineages from cis-Andean hydrographic basins in the present study: two belong to valid species *R. voulezi* and *R. branneri*, and seven divergence lineages currently identified as *R. quelen*. Indeed, many species must be unknown within the *R. quelen* species complex given the wide genus distribution.

Besides the cryptic diversity issue in *R. quelen* complex, we also observed a misidentification problem. Some specimens from the BOLD system (PBRH, BRH FARG, and LARI projects) were taxonomically identified as *R. quelen*, however, they were clustered with Iguaçu River species (*R. voulezi* and *R. branneri*), forming well-defined and monophyletic MOTUs. Although *R. branneri* and *R. voulezi* are considered endemic to the Iguaçu River, our results corroborate with Ríos et al. (2020), indicating that these species’ distribution area may be expanded, with a need for a review of the taxonomic status of both species. Given our results, the revision by Silfvergrip (1996) of the *Rhamdia* is not appropriate to recover *R. quelen* as a natural unit in terms of species phylogenetic concept. 

With rare exceptions (e.g., Iguaçu River basin), we observed that *R. quelen* lineages are specifics from each hydrographic system, i. e., they are not occurring in sympatry. Because these basins were separated a long time ago, the allopatric distribution could reinforce the hypothesis that these lineages represent potential species. Thus, we flag seven independent evolutionary lineages that require further analysis, mainly based on morphological and molecular data, to test the recognition of valid species.

### Allopatric events played an essential role in the lineages diversification processes

The origin and maintenance of the tremendous Neotropical ichthyofauna diversity result from significant historical changes experimented by the continent in the last 90 Mya. The uplift of the Andes and other geological structures, river course changes, and repeated marine incursions and regressions have produced numerous allopatric diversifications in freshwater fishes based on vicariance and geodispersion events (Albert and Reis, 2011). In the following, we will discuss how these events were fundamental to the *Rhamdia* lineages’ divergence.

In our dating analysis, the first diversification event corresponded to the divergence between cis-Andean and Mesoamerican lineages (node 1 - Figure 2A). According to Perdices et al. (2002), Mesoamerican linages resulted from trans-Andean populations’ geodispersion events, already differentiated from the cis-Andean portion, which was promoted after the formation of the Isthmus of Panama. Although this node is calibrated, gaps in *Rhamdia* sampling can lead to our misinterpretation. *Rhamdia* samples from Colombian trans-Andean basins (e.g., Magdalena basin) would give better results about the diversification process of *Rhamdia* MRCA that was probably present in the paleo-Amazonas-Orinoco basin. 

In the cis-Andean clade, the first cladogenetic event (node 5 - Figure 2A) correspond to the divergence between the Northwest group (lineages from Amazonas, Essequibo and Paraguay, and Maranhense Gulf) and Southeast group (Upper and Lower Paraná, Upper and Middle Uruguay, Iguaçu, São Francisco, and coastal basins). This split was dated at 19.2 Ma (95% HPD = 9.1-31.9 Mya) and could be related to successive geodispersion events from paleo-Amazonas-Orinoco (possible ancestral region) to Paleo-Paraná (Brazilian Shield basins). 

Within the Northwest clade, our analysis suggests a cladogenetic event to have happened 14.8 Mya (95% HPD = 5.5-21.6), splitting the MOTU Paraguay and MOTUs from Essequibo, Ucayali (Amazon), and Itapecuru (Maranhense Gulf) basins (Node 6 - Figure 2A). The lineage from the Paraguay basin (MOTU Paraguay), although part of the La Plata basin, was very divergent from the lineages of its main forming rivers (e.g., Paraná, Iguaçu, Uruguay). The mean genetic distance between them was 5.77% (Table 1), values corresponding to species level differentiation for Neotropical fish (Usso et al., 2019). The differentiation of MOTU Paraguay from the MRCA Northwest clade probably was through a geodispersal event from northwestern South America to Paleo-Paraguay. The aquatic fauna exchanges between these paleobasins are commonly reported in various fish groups (Montoya-Burgos, 2003; Sivasundar et al., 2001; Tagliacollo et al., 2015) and was possible until the emergence of an impermeable barrier (Michicola Arch, ~10 Ma - Lundberg, 1998). 

This clade’s next diversification process occurred between Ucayali (Amazon), Essequibo basin, and Maranhense Gulf. The cladogenesis was dated at 7.97 Mya (95% HPD = 3.3-13.5, Node 7 - Figure 2A) and may have been caused by allopatric diversification. It is not possible to conclude whether the promoter event was vicariance (the ancestor spread throughout the northwest region of the continent and was isolated in the Essequibo basin with the reconfiguration of the current drainage system, which led to the modern lineage) or geodispersion (only after reconfiguration of the drainage system the ancestor reached the Essequibo basin). The first proposal seems to be more plausible since the Essequibo basin’s geological history shows connections with both the Orinoco and the Amazon (Branco River) basins (Lujan and Armbruster, 2011).

The split between the MOTUs Itapecuru and Essequibo (Node 8 - Figure 2A), although unsupported (posterior probability <0.90), is probably resulted from a geodispersion event. The divergence may be related to the low sea level during the Miocene-Pliocene that allowed the ichthyofauna to geodisperse among coastal palaeodrainages. This pattern is observed in *Hypostomus* (Montoya-Burgos, 2003), *Serrasalmus* (Hubert et al., 2007), and *Salminus* species (Machado et al., 2018).

The phylogenetic pattern observed in the Southeast clade is typical in multiple geodispersion events caused by headwater captures or marine regressions, both recurrent in the Brazilian Shield basins and coastal region during the Plio-Pleistocene (Tagliacollo et al., 2015; Thomaz et al., 2015). Our analysis placed the MRCA of MOTU 1 (widely distributed in Upper Paraná, Uruguay, and São Francisco basins) and MOTU 2 from Itajaí-Açu basin nearly at 4 Mya (95% HPD = 1.5-6.96). The diversification process between them is probably associate with the erosion of the Serra Geral escarpment, a water divider among three hydrographic basins of southern Brazil: Itajaí-Açu, Paraná (Iguaçu), and Uruguay. According to Sordi (2018), the differential erosion by the Itajaí-Açu river lowered the relief allowing the headwater captures belonging to the inland hydrographic basins (Paraná and Uruguay). This episode allowed the ancestor (node 12 - Figure 2A) to geodisperse into the Itajaí-Açu region.

Finally, the clade formed by MOTU Paraíba do Sul and the *R. branneri* and *R. voulezi* species, whose ancestor is located at node 10 (Figure 2A), has a complex diversification history based on the reorganization of hydrogeological systems resulting from tectonic reactivations and sea-level oscillations. With specimens inhabiting inland (Iguaçu and Lower Paraná) and coastal (North (Paraíba do Sul and Mucuri) and Southern (Itapocu and Lagoa do Peri) coastal) basins (Figure S1), the non-monophyletic distribution pattern in the Bayesian topology reinforce successive geodispersion events (Figure 2A). There are two possible scenarios to explain the diversification processes based on geodispersion events. The first one involves changes in the Iguaçu paleo-river route, which possessed an eastward draining direction (towards the Atlantic Ocean - Albert and Carvalho, 2011). According to the last glacial maximum model proposed by Thomaz et al. (2015), paleoconnections between basins due to low sea level played an essential role in the dispersion of specimens between Brazilian coastal basins, which would explain our data. Probably, the colonization of this region occurred only with the establishment of the current course of Iguaçu to the Middle/Upper Paraná River, as observed for the *Crenicichla lacustris* species complex (Piálek et al., 2012).

Another possible diversification scenario of these three lineages encompasses the diversification events post-redirection of the Iguaçu River course. At least two events may have occurred: the headwater capture from Iguaçu River to coastal basins (Itapocu basin) followed by dispersal routes via coastal paleodrainages.

## Conclusions

Vicariance and geodispersion events played a crucial role in the current intricate distribution pattern of *Rhamdia* lineages from the cis-Andean region, and the interaction between these events likely formed the lineage diversity of this genus. Although not all cis-Andean basins were sampled, the results corroborate that *R. quelen* is a species complex and comprises at least seven potential new species that must be taxonomically analyzed to assess whether they will be recovered or described. Despite the tremendous genetic differentiation between the different lineages, the *Rhamdia* species have similarities in body shape and color pattern, making the identification by non-specialists difficult. The lineages delimitation in the different basins allows minimizing identification errors, avoiding unintentional crossing between species, which may occur with restocking and aquaculture activities since it is a species target for fishery and widely cultivated in southern Brazil, Uruguay and Argentina. Beyond that, it also provides a broad picture of the *Rhamdia* complex distribution in the Neotropical region.

## Supplementary material

The following online material is available for this article:

Table S1 - Lineage, taxon, voucher, locality information and BOLD accession numbers of the analyzed specimens of *Rhamdia*.

Figure S1 - Bayesian inference topology of *Rhamdia* based on COI sequences.

Figure S2 - Time-calibrated topology of *Rhamdia* and outgroups (*Pimelodella*, *Phractocephalus,* and *Steindachneridion*) based on COI sequences.
