# Supplementary material for: Cryptic diversity and diversification processes in three cis-Andean Rhamdia species (Siluriformes: Heptapteridae) revealed by DNA barcoding
Source: Genet Mol Biol. 2021 Jul 12;44(3):e20200470. doi: 10.1590/1678-4685-GMB-2020-0470 (PMC8276235; doi:10.1590/1678-4685-GMB-2020-0470)
Supplement: Table S1 - [file 1415-4757-GMB-44-3-e20200470-s1.pdf]

Genetics and Molecular Biology

https://doi.org/10.1590/1678-4685-GMB-2020-0470

Supplementary Material to "Cryptic diversity and diversification processes in three cis-Andean *Rhamdia* species (Siluriformes: Heptapteridae) revealed by DNA barcoding"

Table S1 - Lineage, taxon, voucher, locality information and BOLD accession numbers of the analyzed specimens of *Rhamdia*.

Prior ID/BOLD	BOLD or Genbank code	Lat	Long	Lineage	Voucher/Museum ID	Locality	River	Basin	Country
R. quelen	PBRH122-19	-27.263	-54.054	MOTU 1	MZUEL 17934	Esperança do Sul	Uruguay	Middle Uruguay	Brazil
R. quelen	PBRH123-19	-27.263	-54.054	MOTU 1	MZUEL 17934	Esperança do Sul	Uruguay	Middle Uruguay	Brazil
R. quelen	PBRH124-19	-27.263	-54.054	MOTU 1	MZUEL 17934	Esperança do Sul	Uruguay	Middle Uruguay	Brazil
R. quelen	PBRH125-19	-27.263	-54.054	MOTU 1	MZUEL 17934	Esperança do Sul	Uruguay	Middle Uruguay	Brazil
R. quelen	PBRH126-19	-27.263	-54.054	MOTU 1	MZUEL 17934	Esperança do Sul	Uruguay	Middle Uruguay	Brazil
R. quelen	PBRH127-19	-27.263	-54.054	MOTU 1	MZUEL 17934	Esperança do Sul	Uruguay	Middle Uruguay	Brazil
R. quelen	PBRH128-19	-27.263	-54.054	MOTU 1	MZUEL 17934	Esperança do Sul	Uruguay	Middle Uruguay	Brazil
R. quelen	PBRH129-19	-27.263	-54.054	MOTU 1	MZUEL 17934	Esperança do Sul	Uruguay	Middle Uruguay	Brazil
R. quelen	PBRH130-19	-27.263	-54.054	MOTU 1	MZUEL 17934	Esperança do Sul	Uruguay	Middle Uruguay	Brazil
R. quelen	PBRH131-19	-27.263	-54.054	MOTU 1	MZUEL 17934	Esperança do Sul	Uruguay	Middle Uruguay	Brazil
R. quelen	PBRH132-19	-27.263	-54.054	MOTU 1	MZUEL 17934	Esperança do Sul	Uruguay	Middle Uruguay	Brazil
R. quelen	PBRH133-19	-27.263	-54.054	MOTU 1	MZUEL 17934	Esperança do Sul	Uruguay	Middle Uruguay	Brazil
R. quelen	PBRH139-19	-27.521	-51.778	MOTU 1	MZUEL 18067	Piratuba	Uruguay	Upper Uruguay	Brazil
R. quelen	PBRH140-19	-27.521	-51.778	MOTU 1	MZUEL 18067	Piratuba	Uruguay	Upper Uruguay	Brazil
R. quelen	PBRH141-19	-27.521	-51.778	MOTU 1	MZUEL 18067	Piratuba	Uruguay	Upper Uruguay	Brazil

Prior ID/BOLD	BOLD or Genbank code	Lat	Long	Lineage	Voucher/Museum ID	Locality	River	Basin	Country
R. quelen	PBRH142-19	-27.521	-51.778	MOTU 1	MZUEL 18067	Piratuba	Uruguay	Upper Uruguay	Brazil
R. quelen	PBRH143-19	-27.521	-51.778	MOTU 1	MZUEL 18067	Piratuba	Uruguay	Upper Uruguay	Brazil
R. quelen	PBRH144-19	-27.521	-51.778	MOTU 1	MZUEL 18067	Piratuba	Uruguay	Upper Uruguay	Brazil
R. quelen	PBRH145-19	-27.521	-51.778	MOTU 1	MZUEL 18067	Piratuba	Uruguay	Upper Uruguay	Brazil
R. quelen	PBRH146-19	-27.521	-51.778	MOTU 1	MZUEL 18067	Piratuba	Uruguay	Upper Uruguay	Brazil
R. quelen	PBRH147-19	-27.521	-51.778	MOTU 1	MZUEL 18067	Piratuba	Uruguay	Upper Uruguay	Brazil
R. quelen	PBRH148-19	-27.521	-51.778	MOTU 1	MZUEL 18067	Piratuba	Uruguay	Upper Uruguay	Brazil
R. quelen	PBRH149-19	-27.521	-51.778	MOTU 1	MZUEL 18067	Piratuba	Uruguay	Upper Uruguay	Brazil
R. quelen	PBRH150-19	-27.521	-51.778	MOTU 1	MZUEL 18067	Piratuba	Uruguay	Upper Uruguay	Brazil
R. quelen	BSB006-10	-19.878	-45.435	MOTU 1	NI	Moema	São Francisco	São Francisco	Brazil
R. quelen	BSB025-10	-19.385	-43.659	MOTU 1	NI	Santana do Riacho	São Francisco	São Francisco	Brazil
R. quelen	BSB062-10	-19.34	-43.638	MOTU 1	NI	Santana do Riacho	São Francisco	São Francisco	Brazil
R. quelen	BSB108-10	-18.725	-44.352	MOTU 1	NI	Corrego Santo Antonio	São Francisco	São Francisco	Brazil
R. quelen	BSB209-10	-19.468	-43.868	MOTU 1	NI	Rio Jaboticatubas	São Francisco	São Francisco	Brazil
R. quelen	BSB377-10	-17.984	-44.169	MOTU 1	NI	Rio Curimatai	São Francisco	São Francisco	Brazil
R. quelen	FUPR777-09	-17.099	-48.761	MOTU 1	35805	Ribeirão Arapuca-Goias	Parana	Upper Parana	Brazil
R. quelen	FUPR778-09	-17.099	-48.761	MOTU 1	35806	Ribeirão Arapuca-Goias	Parana	Upper Parana	Brazil
R. quelen	FUPR779-09	-17.099	-48.761	MOTU 1	35807	Ribeirão Arapuca-Goias	Parana	Upper Parana	Brazil
R. quelen	KU845687.1	-23.936	-51.657	MOTU 1	MZUEL14361-4607	Ivaí River	Parana	Upper Parana	Brazil
R. quelen	KU845688.1	-23.936	-51.657	MOTU 1	MZUEL14361-4607	Ivaí River	Parana	Upper Parana	Brazil
R. quelen	KU845689.1	-23.936	-51.657	MOTU 1	MZUEL14361-4607	Ivaí River	Parana	Upper Parana	Brazil
R. quelen	PBRH121-19	-27.263	-54.054	MOTU 1	MZUEL 17934	Esperança do Sul	Uruguay	Uruguay	Brazil
R. quelen	PBRH117-17	-26.778	-49.386	MOTU 2	MZUEL 18113	Benedito Novo	Benedito Novo	Itajai-Açu	Brazil
R. quelen	PBRH119-17	-26.778	-49.386	MOTU 2	MZUEL 18113	Benedito Novo	Benedito Novo	Itajai-Açu	Brazil

Prior ID/BOLD	BOLD or Genbank code	Lat	Long	Lineage	Voucher/Museum ID	Locality	River	Basin	Country
R. quelen	PBRH118-17	-26.778	-49.386	MOTU 2	MZUEL 18113	Benedito Novo	Benedito Novo	Itajai-Açu	Brazil
R. quelen	PBRH134-19	-27.746	-48.527	MOTU 3	MZUEL 18114	Florianópolis	Peri Lagoon	Peri Lagoon	Brazil
R. quelen	PBRH135-19	-27.746	-48.527	MOTU 3	MZUEL 18114	Florianópolis	Peri Lagoon	Peri Lagoon	Brazil
R. quelen	PBRH136-19	-27.746	-48.527	MOTU 3	MZUEL 18114	Florianópolis	Peri Lagoon	Peri Lagoon	Brazil
R. quelen	PBRH137-19	-27.746	-48.527	MOTU 3	MZUEL 18114	Florianópolis	Peri Lagoon	Peri Lagoon	Brazil
R. quelen	PBRH138-19	-27.746	-48.527	MOTU 3	MZUEL 18114	Florianópolis	Peri Lagoon	Peri Lagoon	Brazil
R. quelen	PBRH152-19	-27.746	-48.527	MOTU 3	MZUEL 18112	São Francisco do Sul	Itapocu	Itapocu	Brazil
R. quelen	PBRH153-19	-27.746	-48.527	MOTU 3	MZUEL 18112	São Francisco do Sul	Itapocu	Itapocu	Brazil
R. voulezi	BRH013-16	-25.621	-52.620	MOTU 3	MZUEL 16503	Salto Osório Reservoir	Iguaçu	Iguaçu	Brazil
R. voulezi	BRH014-16	-25.621	-52.620	MOTU 3	MZUEL 16503	Salto Osório Reservoir	Iguaçu	Iguaçu	Brazil
R. voulezi	BRH015-16	-25.621	-52.620	MOTU 3	MZUEL 16503	Salto Osório Reservoir	Iguaçu	Iguaçu	Brazil
R. voulezi	BRH016-16	-25.621	-52.620	MOTU 3	MZUEL 16503	Salto Osório Reservoir	Iguaçu	Iguaçu	Brazil
R. voulezi	BRH017-16	-25.621	-52.620	MOTU 3	MZUEL 16503	Salto Osório Reservoir	Iguaçu	Iguaçu	Brazil
R. voulezi	BRH018-16	-25.621	-52.620	MOTU 3	MZUEL 16503	Salto Osório Reservoir	Iguaçu	Iguaçu	Brazil
R. voulezi	BRH019-16	-25.621	-52.620	MOTU 3	MZUEL 16503	Salto Osório Reservoir	Iguaçu	Iguaçu	Brazil
R. quelen	PBRH151-19	-26.251	-48.615	MOTU 4	NI	São Francisco do Sul	Itapocu River	Itapocu	Brazil
R. quelen	FARG317-07	-37.32	-57.19	MOTU 4	INIDEP-T 0316	Mar Chiquita coastal lagoon	Paraná	Lower Paraná	Argentina
R. quelen	FARGB235-11	-38.335	-61.601	MOTU 4	UNMDP-T 0403	El Divisorio stream	Paraná	Lower Paraná	Argentina
R. quelen	FARGB236-11	-38.335	-61.601	MOTU 4	UNMDP-T 0404	El Divisorio stream	Paraná	Lower Paraná	Argentina
R. quelen	FARGB250-11	-38.335	-61.601	MOTU 4	UNMDP-T 0418	El Divisorio stream	Paraná	Lower Paraná	Argentina
R. quelen	FARGB297-11	-34.082	-61	MOTU 4	UNMDP T 465	Tributario rio Rojas (Ascencion)	Paraná	Lower Paraná	Argentina
R. quelen	FARGB365-11	-33.317	-60.223	MOTU 4	UNMDP T 533	Rio Parana (San Nicolas)	Paraná	Lower Paraná	Argentina
R. quelen	LARI368-13	-32.903	-60.653	MOTU 4	NI	Laguna el saco	Paraná	Lower Paraná	Argentina
R. branneri	BRH001-16	-25.621	-52.620	MOTU 4	MZUEL 16504	Salto Osório Reservoir	Iguaçu	Iguaçu	Brazil
R. branneri	BRH002-16	-25.621	-52.620	MOTU 4	MZUEL 16504	Salto Osório Reservoir	Iguaçu	Iguaçu	Brazil

Prior ID/BOLD	BOLD or Genbank code	Lat	Long	Lineage	Voucher/Museum ID	Locality	River	Basin	Country
R. branneri	BRH003-16	-25.621	-52.620	MOTU 4	MZUEL 16504	Salto Osório Reservoir	Iguaçu	Iguaçu	Brazil
R. branneri	BRH004-16	-25.621	-52.620	MOTU 4	MZUEL 16504	Salto Osório Reservoir	Iguaçu	Iguaçu	Brazil
R. branneri	BRH005-16	-25.621	-52.620	MOTU 4	MZUEL 16504	Salto Osório Reservoir	Iguaçu	Iguaçu	Brazil
R. branneri	BRH006-16	-25.621	-52.620	MOTU 4	MZUEL 16504	Salto Osório Reservoir	Iguaçu	Iguaçu	Brazil
R. branneri	BRH007-16	-25.621	-52.620	MOTU 4	MZUEL 16504	Salto Osório Reservoir	Iguaçu	Iguaçu	Brazil
R. branneri	BRH008-16	-25.621	-52.620	MOTU 4	MZUEL 16504	Salto Osório Reservoir	Iguaçu	Iguaçu	Brazil
R. branneri	BRH009-16	-25.621	-52.620	MOTU 4	MZUEL 16504	Salto Osório Reservoir	Iguaçu	Iguaçu	Brazil
R. branneri	BRH010-16	-25.621	-52.620	MOTU 4	MZUEL 16504	Salto Osório Reservoir	Iguaçu	Iguaçu	Brazil
R. branneri	BRH011-16	-25.621	-52.620	MOTU 4	MZUEL 16504	Salto Osório Reservoir	Iguaçu	Iguaçu	Brazil
R. branneri	BRH012-16	-25.621	-52.620	MOTU 4	MZUEL 16504	Salto Osório Reservoir	Iguaçu	Iguaçu	Brazil
R. quelen	MUCU033-13	-17.743	-40.794	MOTU 5	MCNIP-1431	Rio Mucuri	Mucuri	Mucuri	Brazil
R. quelen	MUCU083-13	-17.897	-40.685	MOTU 5	MCNIP-1433	Rio Mucuri	Mucuri	Mucuri	Brazil
R. quelen	FPSR094-09	-22.637	-44.616	MOTU 5	29356	Sao Paulo	Paraiba do Sul	Paraíba do Sul	Brazil
R. quelen	FPSR095-09	-22.637	-44.616	MOTU 5	29357	Sao Paulo	Paraiba do Sul	Paraíba do Sul	Brazil
R. quelen	FPSR096-09	-22.637	-44.616	MOTU 5	29358	Sao Paulo	Paraiba do Sul	Paraíba do Sul	Brazil
R. quelen	FPSR097-09	-22.637	-44.616	MOTU 5	29359	Sao Paulo	Paraiba do Sul	Paraíba do Sul	Brazil
R. quelen	FPSR098-09	-22.637	-44.616	MOTU 5	29360	Sao Paulo	Paraiba do Sul	Paraíba do Sul	Brazil
R. quelen	GBMIN119705-17	NI	NI	MOTU 6	NI	Peruan Amazon	Ucayali	Amazon	Peru
Rhamdia sp.	GBMIN124598-17	NI	NI	MOTU 6	NI	Peruan Amazon	Ucayali	Amazon	Peru
Rhamdia sp.	GBMIN124597-17	NI	NI	MOTU 7	NI	Peruan Amazon	Ucayali	Amazon	Peru
R. quelen	ITAPE403-15	-2.94	-44.36	MOTU 7	NI	Rosario, Maranhão	Itapecuru	Maranhense Gulf	Brazil
R. quelen	ITAPE404-15	-2.94	-44.36	MOTU 7	NI	Rosario, Maranhão	Itapecuru	Maranhense Gulf	Brazil
R. quelen	TZGAA005-06	7.5	-59.55	MOTU 8	NI	Chinese Landing, at Store	Esequibo	Orinoco	Guiana
R. quelen	TZGAA016-06	7.5	-59.55	MOTU 8	NI	Chinese Landing, at Store	Esequibo	Orinoco	Guiana

Prior ID/BOLD	BOLD or Genbank code	Lat	Long	Lineage	Voucher/Museum ID	Locality	River	Basin	Country
R. quelen	TZGAA027-06	7.5	-59.55	MOTU 8	NI	Chinese Landing, at Store	Esequibo	Orinoco	Guiana
R. quelen	TZGAA051-06	7.5	-59.55	MOTU 8	NI	Chinese Landing, at Store	Esequibo	Orinoco	Guiana
R. quelen	TZGAA063-06	7.5	-59.55	MOTU 8	NI	Chinese Landing, at Store	Esequibo	Orinoco	Guiana
R. quelen	TZGAA075-06	7.65	-59.3	MOTU 8	NI	Purchased at Santa Cruz	Esequibo	Orinoco	Guiana
R. quelen	TZGAA087-06	7.65	-59.3	MOTU 8	NI	Purchased at Santa Cruz	Esequibo	Orinoco	Guiana
R. quelen	TZGAA088-06	7.5	-59.55	MOTU 8	NI	Chinese Landing, at Store	Esequibo	Orinoco	Guiana
R. quelen	KU845690.1	-19.523	-57.040	MOTU 9	MZUEL14361-4607	Miranda river	Paraguay	Upper Paraguay	Brazil
R. quelen	KU845691.1	-19.523	-57.040	MOTU 9	MZUEL14361-4607	Miranda river	Paraguay	Upper Paraguay	Brazil
R. quelen	KU845692.1	-19.523	-57.040	MOTU 9	MZUEL14361-4607	Miranda river	Paraguay	Upper Paraguay	Brazil
R. quelen	KU845693.1	-19.523	-57.040	MOTU 9	MZUEL14361-4607	Miranda river	Paraguay	Upper Paraguay	Brazil
R. quelen	KU845694.1	-19.523	-57.040	MOTU 9	MZUEL14361-4607	Miranda river	Paraguay	Upper Paraguay	Brazil
R. laticauda	BSFFA418-07	8.764	-82.827	*R. laticauda*	BSFFA418-07	Chiquiri	Chiriqui Viejo	Panama	Panama
R. laticauda	BSFFA459-07	9.040	-82.285	*R. laticauda*	STRI-00821	Bocas	Robalo	Panama	Panama
R. laticauda	BSFFA462-07	12.012	-84.668	*R. laticauda*	STRI-06328	San Juan	Espavel	Nicaragua	Nicaragua
R. laticauda	BSFFA893-13	9.068	-82.292	*R. laticauda*	STRI-00740	Bocas	Quebrada La NI Fe	Panama	Panama
R. quelen	BSFFA450-07	9.3160	-78.686	MOTU Mesoamerican 1	STRI-00849	Tuira	Aguas Claras	Panama	Panama
R. quelen	BSFFA454-07	8.1125	-77.112	MOTU Mesoamerican 1	STRI-00780	Tuira	Yape NI	Panama	Panama
R. quelen	BSFFA457-07	8.9794	-78.505	MOTU Mesoamerican 1	STRI-00770	Tuira	Ipeti	Panama	Panama
R. quelen	BSFFA813-07	8.1351	-80.576	MOTU Mesoamerican 1	STRI-00807	Herrera	Santa Maria	Panama	Panama
R. quelen	BSFFA873-13	8.135	-80.576	MOTU Mesoamerican 1	STRI-00808	Santa Maria	Santa Maria	Panama	Panama
R. quelen	BSFFA883-13	8.618	-77.831	MOTU Mesoamerican 1	STRI-00829	Tuira	Membrillo NI	Panama	Panama
R. quelen	BSFFA812-07	12.012	-84.668	MOTU Mesoamerican 2	STRI-06328	San Juan	Espavel	Nicaragua	Nicaragua
R. quelen	BSFFA814-07	12.945	-86.845	MOTU Mesoamerican 2	STRI-06339	Estero Real-Tempisque	Hato Grande	Nicaragua	Nicaragua

Prior ID/BOLD	BOLD or Genbank code	Lat	Long	Lineage	Voucher/Museum ID	Locality	River	Basin	Country
R. guatemalensis	HBGM265-14	20.734	-87.19	*R. guatemalensis*	P6318	Benedito Juarez	Predio Delirios	Mexico	Mexico
R. guatemalensis	HBGM266-14	20.734	-87.19	*R. guatemalensis*	P6318	Benedito Juarez	Predio Delirios	Mexico	Mexico
R. guatemalensis	HBGM267-14	20.734	-87.19	*R. guatemalensis*	P6318	Benedito Juarez	Predio Delirios	Mexico	Mexico

NI: Nonexistent information
