# Supplementary material for: Cryptic diversity and diversification processes in three cis-Andean Rhamdia species (Siluriformes: Heptapteridae) revealed by DNA barcoding
Source: Genet Mol Biol. 2021 Jul 12;44(3):e20200470. doi: 10.1590/1678-4685-GMB-2020-0470 (PMC8276235; doi:10.1590/1678-4685-GMB-2020-0470)
Supplement: Figure S1 - [file 1415-4757-GMB-44-3-e20200470-s2.pdf]

Genetics and Molecular Biology

https://doi.org/10.1590/1678-4685-GMB-2020-0470

Supplementary Material to "Cryptic diversity and diversification processes in three cis-Andean *Rhamdia* species (Siluriformes: Heptapteridae) revealed by DNA barcoding"

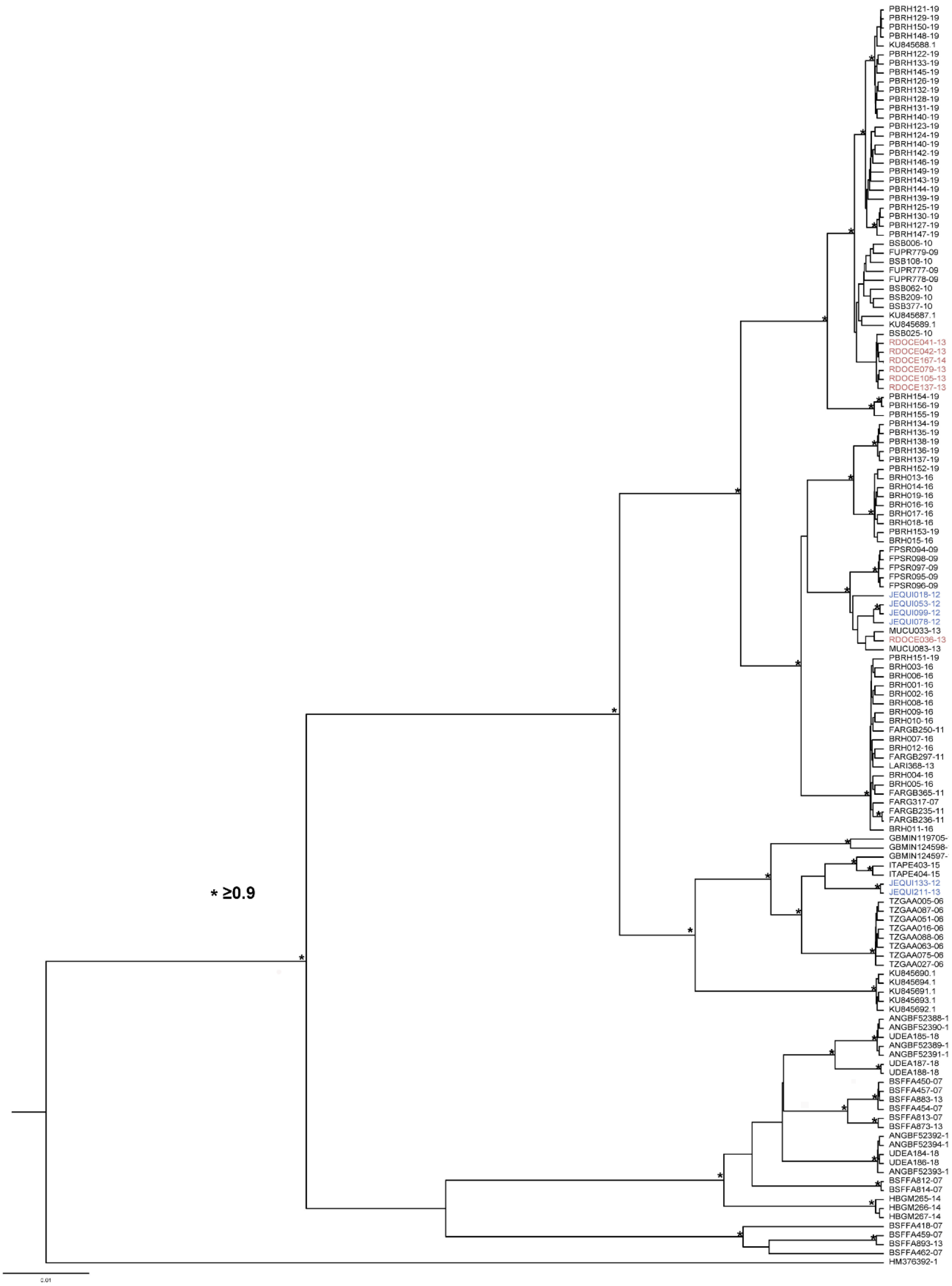

Figure S1 - Bayesian inference topology of *Rhamdia* based on COI sequences included sequences from Jequitinhonha (blue and red colors). The asterisks represent posterior probabilities above 0.9.
