# Supplementary material for: Cryptic diversity and diversification processes in three cis-Andean Rhamdia species (Siluriformes: Heptapteridae) revealed by DNA barcoding
Source: Genet Mol Biol. 2021 Jul 12;44(3):e20200470. doi: 10.1590/1678-4685-GMB-2020-0470 (PMC8276235; doi:10.1590/1678-4685-GMB-2020-0470)
Supplement: Figure S2 - [file 1415-4757-GMB-44-3-e20200470-s3.pdf]

Genetics and Molecular Biology

https://doi.org/10.1590/1678-4685-GMB-2020-0470

Supplementary Material to "Cryptic diversity and diversification processes in three cis-Andean *Rhamdia* species (Siluriformes: Heptapteridae) revealed by DNA barcoding"

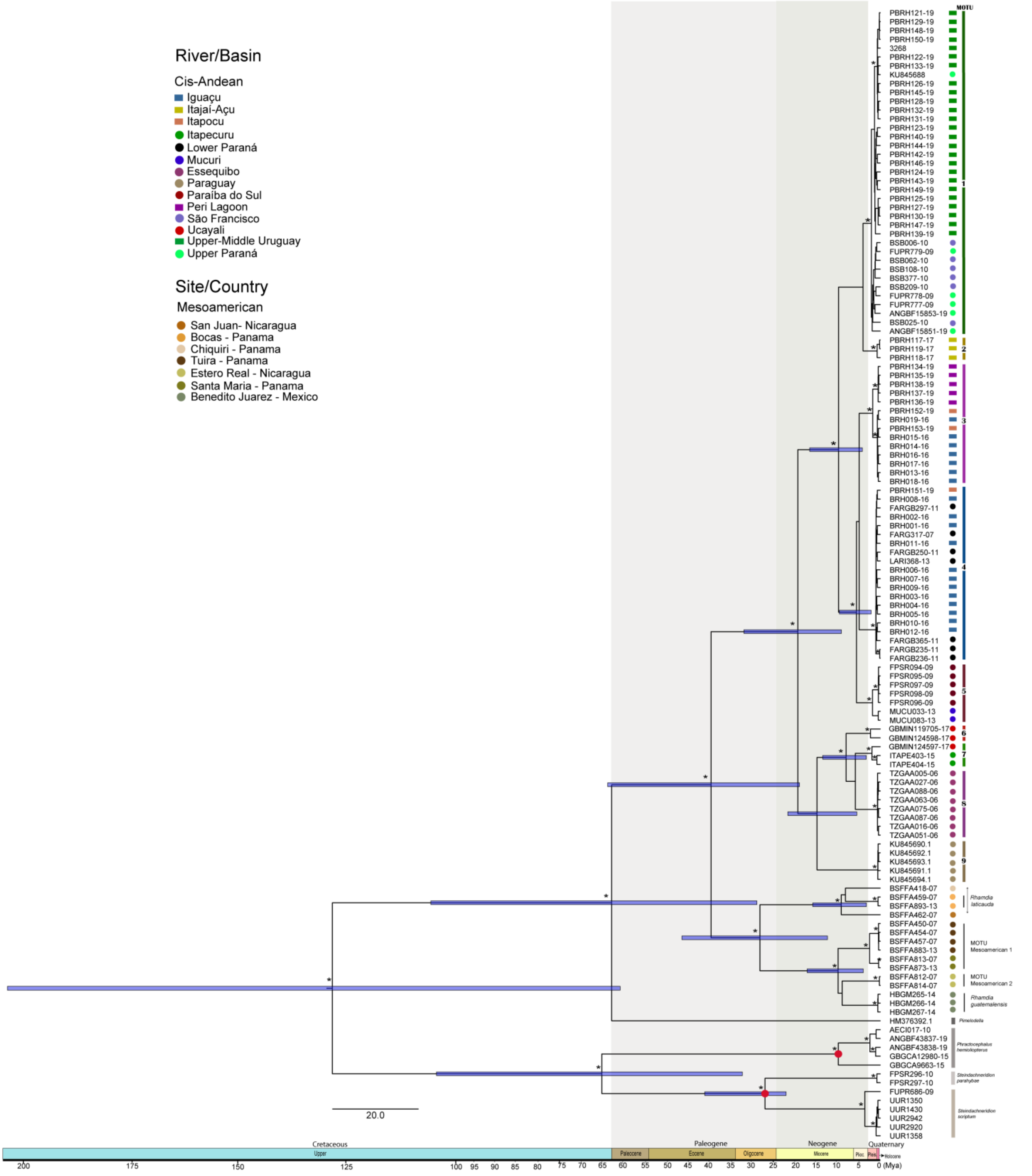

Figure S2 - Time-calibrated topology of *Rhamdia* and outgroups (*Pimelodella*, *Phractocephalus,* and *Steindachneridion*) based on COI sequences. The asterisks represent posterior probabilities above 0.9. Circles represent samples from BOLD System (http://www.boldsystems.org/), while squares are sequences obtained in this study. Red circles indicate the calibration points.
